# Intimate partner violence and mental health—Remarks from two Chief Editors on a joint publishing venture

**DOI:** 10.3402/ejpt.v5.25679

**Published:** 2014-09-12

**Authors:** Miranda Olff, Stig Wall

**Affiliations:** 1 European Journal of Psychotraumatology; 2 Global Health Action

The World Health Organization (WHO) recently estimated that one out of every three women will experience physical or sexual violence by an intimate partner or sexual violence by a non-partner during her lifetime (Abrahams et al., 2014; Devries et al., 2013). Both men and women can be victims as well as perpetrators of IPV, and pregnancy does not prevent it from occurring. Intimate partner violence (IPV) is the most common form of violence against women, and there is a clear link with mental health both as a consequence and as a background etiology. IPV thus affects posttraumatic stress disorder, major depressive disorder, generalized anxiety disorder, risk of suicide, drug and alcohol abuse, while mental health consequences are often comorbid. Not only is mental health affected but also physical health. IPV prevalence has been estimated to be 38% in family medicine and 40% in emergency medicine (Sprague, 2013).

The 2010 Global Burden of Disease Study ranked IPV fifth in terms of years of life lost owing to disability for women. In 2012, for the first time in Sweden, as a sign of the emergence of viewing violence from a public health perspective, a full chapter of the latest National Public Health Report of Sweden was devoted to violence (Health in Sweden, 2012). Despite the overwhelming evidence of magnitude and global reach of IPV, interventions are rare and an appropriate health sector response is called for (Jewkes, 2013). However, a recent Cochrane review concluded that “there is insufficient evidence to justify universal screening for IPV in health care settings” (O'Doherty et al., 2014). In a Declaration on Mental Health in Africa, urgent action is called for to address mental health issues, to include mental health into the post-2015 Sustainable Development Goals and specifically to respond to the mental health needs that arise as a consequence of violence in society, especially against women and children (Daar et al., 2014).

In order to promote a broad perspective on research on IPV and mental health, the editors of the *European Journal of Psychotraumatology* (EJPT) and *Global Health Action* (GHA) have in a unique collaboration created two mutually illuminating series of high-quality state of the art papers on the topic. Guest Editors Mary Ellsberg for *GHA* and Sheila Sprague for *EJPT*, both experts in the field of IPV, have brought cutting edge knowledge together addressing IPV and mental health from a global health and psychotrauma perspective, respectively (Ellsberg & Emmelin, 2014; Sprague et al., 2014). They both have been pioneers in making violence against women visible as a major global health problem during the past two decades.

Professor Ellsberg's PhD thesis from 2000 with the thought-provoking title “Candies in Hell” (Ellsberg, 2000) not only disclosed hitherto unpublished figures on life-time and current prevalence but also contributed to the debate on the reform of the Nicaraguan Criminal Code on sanctions for domestic violence and protection for victims. The study thus called attention at a national level to the magnitude and serious consequences of domestic violence. The work from Nicaragua was also instrumental for developing the methodology used in the forthcoming WHO Multi-Country Study revealing the magnitude of domestic violence around the world (Garcia-Moreno, Jansen, Ellsberg, Heise, & Watts, 2006).

Sheila Sprague's PhD thesis on IPV in orthopedic trauma clinics is one of the first bodies of work on the issue of IPV in surgical fields. The multinational Prevalence of Abuse and Intimate Partner Violence Surgical Evaluation (PRAISE) study was the first to establish the scope of the issue in orthopedic trauma clinics. Dr. Sprague's thesis also highlighted the barriers to IPV screening in orthopedic clinics and other health care fields, and identified surgeons’ and patients’ perceptions of IPV and discussing IPV in orthopedic trauma clinics (Sprague, 2013).

It is against the above background that our two journals jointly issued a Call for Papers on IPV and Mental Health in early 2014. In the Call, we invited papers showing the implications of IPV for mental health, as well as papers analyzing the mental health background. We specifically invited contributions on

1. Mental health issues related to victimization, perpetration, and witnessing of IPV
2. IPV in under-researched populations
3. Intersections between IPV and violence against children
4. Intervention research to prevent or mitigate the effects of IPV, at a structural, community, relational, or individual levels, including health services

We received a total of 25 submissions, 14 of which have been accepted after peer review and are now published in one of both journals. One can identify three different themes among these articles—the interconnection between IPV and mental health, the impact on children, and the health sector response. The research questions of the 14 papers are briefly outlined in the table.

**Table:** 

Research theme	*EJPT* papers	*GHA* papers
IPV & Mental Health	How is IPV related to specific mental health outcomes? (Lagdon, Armour, & Stringer, 2014)	What is the role of mental health in the primary prevention of violence? (Gevers & Dartnall, 2014)
		How does cultural tolerance of IPV influence abuse? (Stockman et al., 2014)
IPV & Children	How does IPV affect longitudinal trajectories and intergenerational transmission of violence? (Armour & Sleath, 2014)	How do women's exposure to IPV and education level influence corporal punishment of children? (Salazar, Dahlblom, Solórzano, & Herrera, 2014)
	Why do women stay in abusive relationships? (Burnette & Cannon, 2014)	How does experience of IPV relate to maternal depression after childbirth? (Kabir, Nasreen, & Edhborg, 2014)
	Is there a link between childhood maltreatment and adult IPV development? (Webermann, Brand, & Chasson, 2014)	
IPV & Health Services	How can IPV be effectively prevented and treated? (Hansen, Eriksen, & Elklit, 2014)	How can a community be mobilized for primary prevention of IPV? (Kyegombe et al., 2014)
	How can women's shelters be helpful? (Hoyeck, Madden, Freeman, Scott, & Bhandari, 2014)	How do stakeholders see the role of health services in addressing mental health impact of IPV? (Chepuka et al., 2014)
	How can the vicious cycle of IPV and drug abuse be broken? (Simonelli, Pasquali, & De Palo, 2014)	How should service providers best address IPV as part of their service delivery? (Rees, Zweigenthal, & Joyner, 2014)

We are pleased to present the two complementary special issues in our two journals. Special attention will be paid to it at the 16th World Congress of Psychiatry in Madrid in September 2014.

*Miranda Olff* Chief Editor European Journal of Psychotraumatology *Stig Wall* Chief Editor Global Health Action
